# Supplementary material for: Technological Quality, Amino Acid and Fatty Acid Profile of Broiler Meat Enhanced by Dietary Inclusion of Black Soldier Fly Larvae
Source: Foods. 2021 Feb 2;10(2):297. doi: 10.3390/foods10020297 (PMC7913093; doi:10.3390/foods10020297)
Supplement: Supplementary file 1 [file foods-10-00297-s001.pdf]

Supplementary material

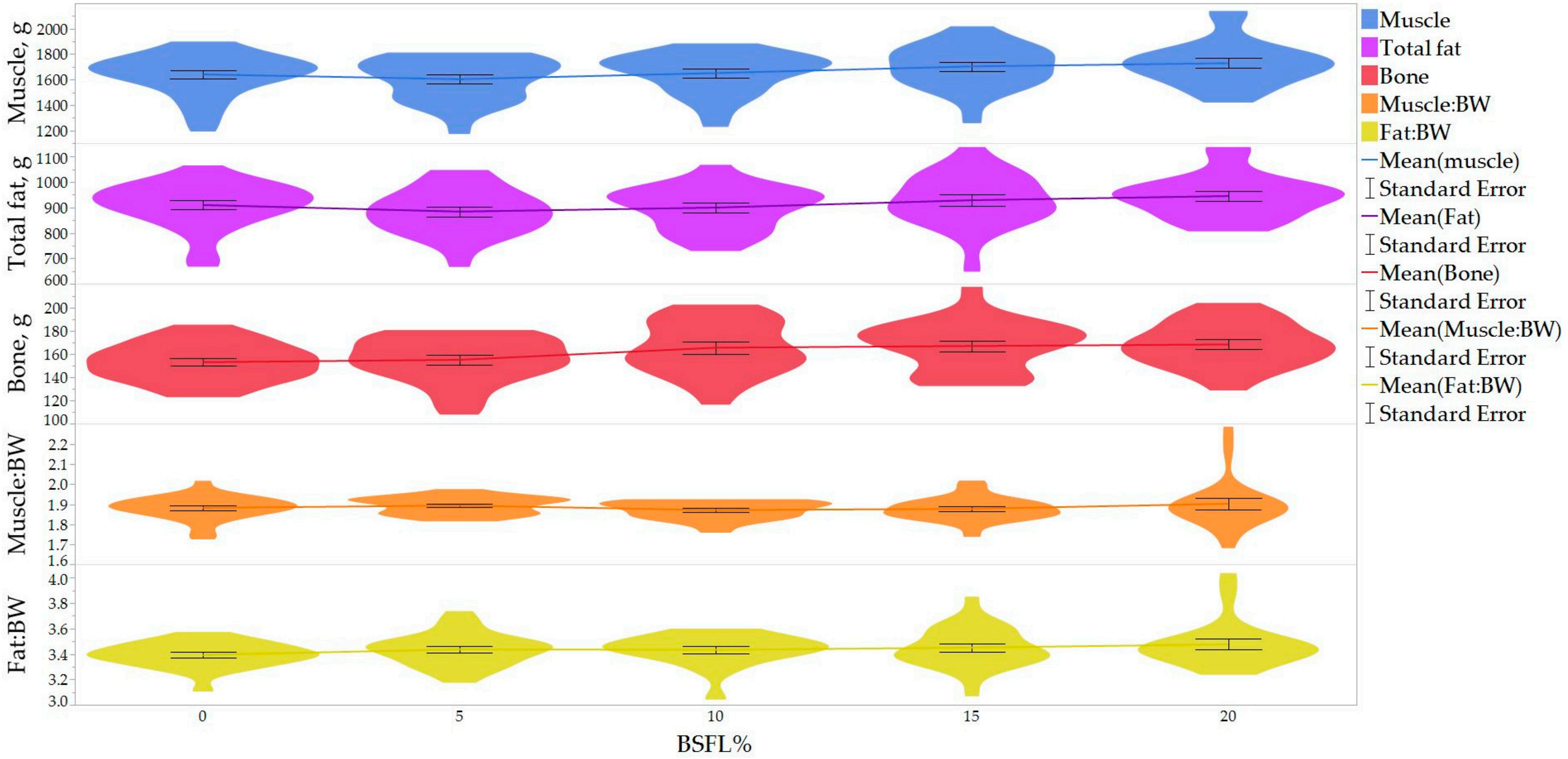

Figure S1*:* Violin plots representing the computed tomography (CT) compositional carcass traits for broilers – Muscle (g) in blue (first row), Total fat (g) in purple (second row), Bone (g) in red (third row), Muscle: Body weight (BW) in yellow (forth row); and Fat: Body Weight (BW) in green (fifth row) – fed diets including the maximum inclusion levels – 0%, 5%, 10%, 15%, and 20% – of Black Soldier Fly larvae (BSFL%) are shown. A trend line for each carcass trait among BSFL% is also shown. Violin plots represent the distribution and density of the data, where thicker areas represent where most observations are. Thinner areas of the violin represent the place where fewer or non-observations exist. BW = the final body weight (in grams) of broilers at day 42.

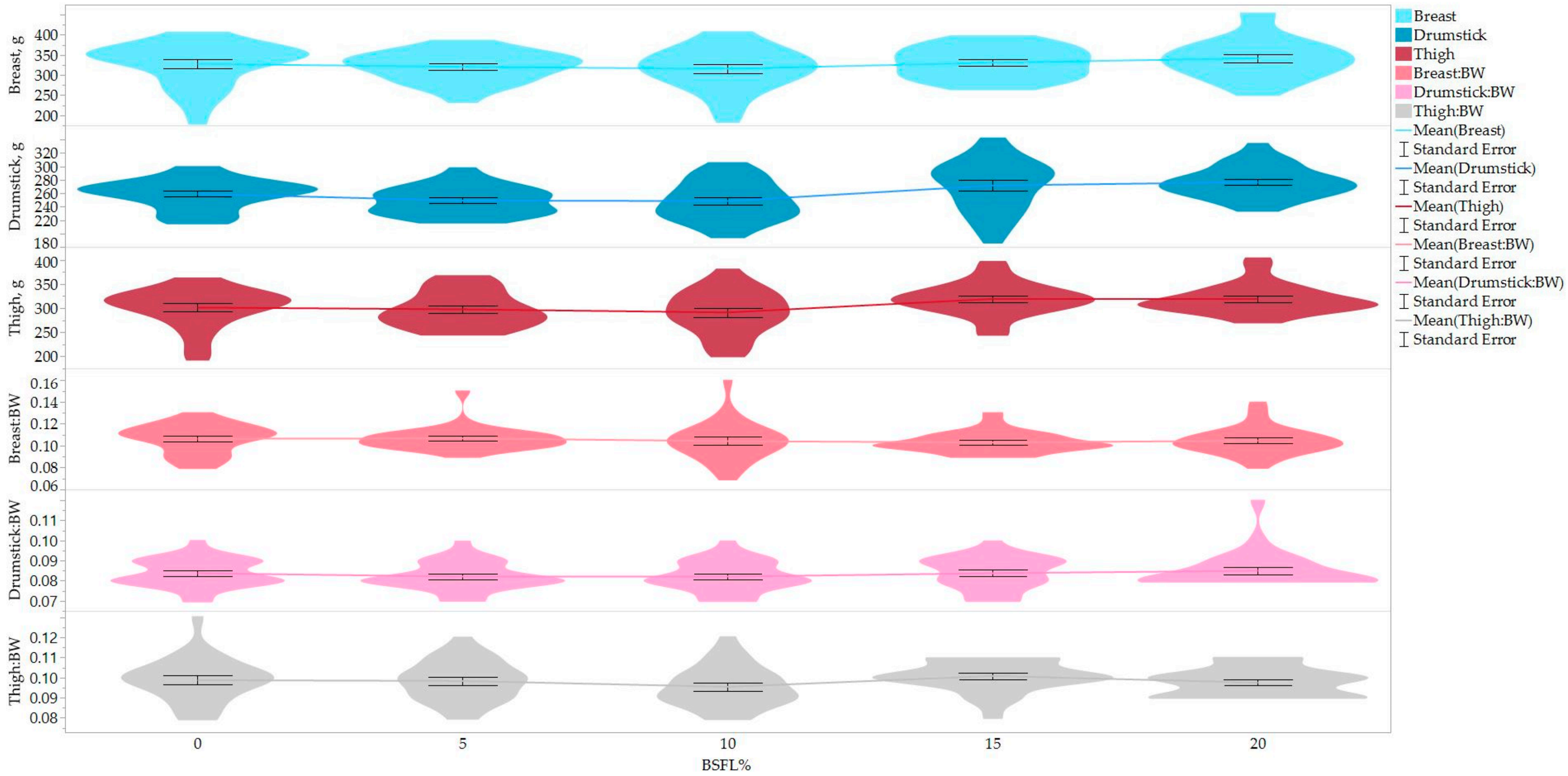

Figure S2: Violin plots representing cut yield traits for broilers – Breast (g) in light blue (first row), Drumstick (g) in dark blue (second row), Thigh (g) in red (third row), Breast: BW ratio in pink (forth row), Drumstick: BW ratio in light pink (fifth row), and Thigh: BW ratio in grey (sixth row) - fed diets including the maximum inclusion levels – 0%, 5%, 10%, 15%, and 20% – of Black Soldier Fly larvae (BSFL%) are shown. A trend line for each carcass trait among BSFL% is also shown. Violin plots represent the distribution and density of the data, where thicker areas represent where most observations are. Thinner areas of the violin represent the place where fewer or non-observations exist. BW = the final body weight (in grams) of broilers at day 42.
